# DeepCAPE: A Deep Convolutional Neural Network for the Accurate Prediction of Enhancers

**DOI:** 10.1016/j.gpb.2019.04.006

**Published:** 2021-02-11

**Authors:** Shengquan Chen, Mingxin Gan, Hairong Lv, Rui Jiang

**Affiliations:** 1 Ministry of Education Key Laboratory of Bioinformatics, Bioinformatics Division at the Beijing National Research Center for Information Science and Technology, Center for Synthetic and Systems Biology, Department of Automation, Tsinghua University, Beijing 100084, China; 2 Department of Management Science and Engineering, School of Economics and Management, University of Science and Technology Beijing, Beijing 100083, China

**Keywords:** Enhancer prediction, Chromatin accessibility, Data integration, Transcription factor binding motif, Disease-associated regulatory element

## Abstract

The establishment of a landscape of enhancers across human cells is crucial to deciphering the mechanism of gene regulation, cell differentiation, and disease development. High-throughput experimental approaches, which contain successfully reported enhancers in typical cell lines, are still too costly and time-consuming to perform systematic identification of enhancers specific to different cell lines. Existing computational methods, capable of predicting regulatory elements purely relying on DNA sequences, lack the power of cell line-specific screening. Recent studies have suggested that **chromatin accessibility** of a DNA segment is closely related to its potential function in regulation, and thus may provide useful information in identifying regulatory elements. Motivated by the aforementioned understanding, we integrate DNA sequences and chromatin accessibility data to accurately predict enhancers in a cell line-specific manner. We proposed DeepCAPE, a deep convolutional neural network to predict enhancers via the integration of DNA sequences and DNase-seq data. Benefitting from the well-designed feature extraction mechanism and skip connection strategy, our model not only consistently outperforms existing methods in the imbalanced classification of cell line-specific enhancers against background sequences, but also has the ability to self-adapt to different sizes of datasets. Besides, with the adoption of auto-encoder, our model is capable of making cross-cell line predictions. We further visualize kernels of the first convolutional layer and show the match of identified sequence signatures and known motifs. We finally demonstrate the potential ability of our model to explain functional implications of putative disease-associated genetic variants and discriminate disease-related enhancers. The source code and detailed tutorial of DeepCAPE are freely available at https://github.com/ShengquanChen/DeepCAPE.

## Introduction

Enhancers are distal regulatory elements that can be bound by transcription factors (TFs) to boost the expression of their target genes. As important regulatory elements, enhancers collaborate with promoters to regulate the transcription of genes in a *cis*-acting manner, receiving more and more attention in studies of cell differentiation [1], human diseases [2], and phenotypic diversity [3]. However, due to such facts as far away from target genes, the absence of common sequence features, and the high cell line specificity, it has long been a challenging task to systematically and precisely identify enhancers in a specific cell line.

Enhancers are usually identified by high-throughput biological experiments. For example, Heintzman and Ren [4] used ChIP-seq experiments to establish a landscape of binding sites for individual TFs; May et al. [5] mapped the binding sites of transcription coactivators p300 and CBP to a large number of enhancers. With the understanding that enhancers are marked by monomethylation of H3K4 [6], genome-wide identification of enhancers has been conducted in large-scale projects such as ENCODE [7] and Roadmap [8]. Besides, using a technique called Cap Analysis of Gene Expression (CAGE), the FANTOM project [9] has mapped promoters and enhancers that are active in mammalian primary cell lines [10]. Considering that experimental approaches are expensive and time-consuming for large-scale identification of enhancers, computational methods have been proposed to predict regulatory elements. For example, kmer-SVM used *k*-mer frequencies of a DNA fragment with a support vector machine (SVM) to classify regulatory elements [11]; gkmSVM and LS-GKM allowed gaps in a *k*-mer and improved the prediction performance [12], [13]; methods based on random forests [14] and decision trees [15] have also been introduced.

Over the past five years, deep learning has been incorporated into bioinformatics studies. For example, DeepBind used a convolutional neural network (CNN) to predict binding proteins and showed higher prediction power than traditional classifiers [16]; DeepSEA learned DNA regulatory codes via a CNN from epigenomic data and predicted effects of non-coding variants [17]; DeepEnhancer predicted enhancers purely relying on DNA sequences and outperformed SVM-based methods [18]; DeepCRISPR unified sgRNA on-target and off-target site prediction into one framework with deep learning [19]. The success of these methods suggests that deep learning is a powerful tool in genomic studies. Nevertheless, these methods, which use only DNA sequence information, obviously lack the power of making predictions in a cell line-specific manner, because DNA sequences are identical in different cell lines.

Chromatin accessibility of the genome has received more and more attention in the recent years. It is known that putative accessible regions in the genome often work with TFs, RNA polymerases, and other cellular machines to regulate gene expression [20]. The development of high-throughput sequencing techniques, such as DNase-seq and Assay for Transposase-Accessible Chromatin with high-throughput sequencing (ATAC-seq), has enabled the accumulation of a vast amount of chromatin profiles across a variety of cell lines and provides a great opportunity to study transcription factor binding sites (TFBSs), DNA methylation sites, histone modification markers, and other regulatory elements [21], [22]. It is therefore natural to integrate DNA sequences and chromatin accessibility information in a single neural network model for the study of cell line-specific enhancers.

With the aforementioned understanding, we propose in this study DeepCAPE, a deep CNN for the accurate prediction of enhancers, using DNA sequences and DNase-seq data. Through comprehensive experiments, we show that our model is not only superior to existing methods in the prediction of enhancers, but also able to predict enhancers across cell lines. With a visualization strategy, we show that sequence motifs discovered by our method successfully match known motifs. Through joint analysis of prediction results with genome-wide association study (GWAS) data, we show the potential ability of our method to identify genetic variants associated with liver cancer and discriminate enhancers related to lymphoma.

## Method

### Data collection and processing

We use the promoter enhancer slider selector tool (PrESSTo) to download experimentally verified enhancers specific to 9 different cell lines from the FANTOM project, including epithelial cell of esophagus, melanocyte, cardiac fibroblast, keratinocyte, myoblast, stromal cell, mesenchymal cell, natural killer cell, and monocyte. We use two strategies to generate negative samples, *i.e.*, non-enhancer fragments that do not overlap with enhancers. First, we randomly sample DNA fragments of variable length from the background genome, with the constraint that the length and GC content of negative samples should be identically distributed as those of known enhancers. The background genome is defined as the entire human reference genome (GRCh37), excluding known enhancers, promoters for coding and non-coding genes, and exonic regions for coding and non-coding genes. Second, we discard the constraint on the GC content to demonstrate the adaptability of our method to different genome contexts. The first model is more stringent and is used throughout this study. We set the ratios of positive to negative samples to 1:10 and 1:20, *i.e.*, for each positive sample, we generate 10 and 20 negative samples, respectively.

**Supplementary Figure S1:**
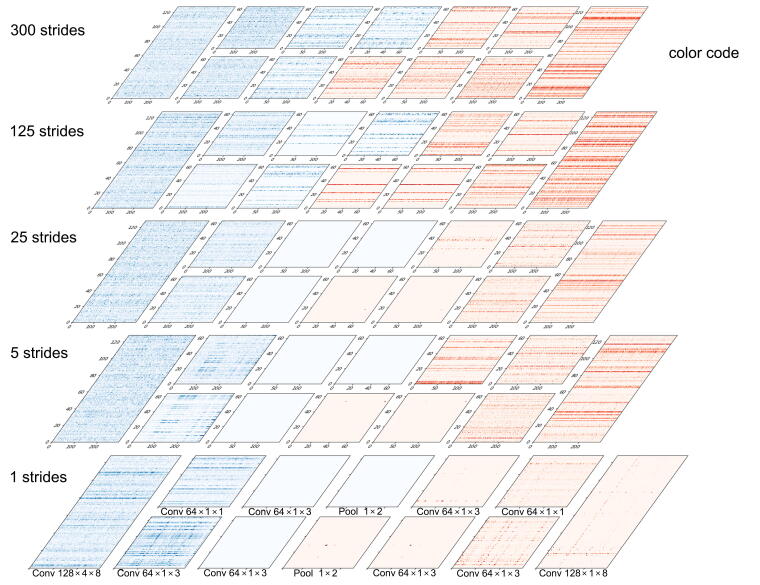
**Visualization of activated features on the merge-layer extracted by DeepCAPE for training sets of different sizes (different augmentation strides)**. The blue and red points represent activation degree of features in DNA and DNase modules, respectively (darker color corresponds to higher activation degree). With abundant training samples (*e.g.*, stride 1), DeepCAPE is inclined to activate only low-level features, which are extracted by the first three convolutional layers. When the sample size is limited (*e.g.*, stride 300), however, DeepCAPE can also activate high-level features, which are extracted by the last three layers.

We download raw sequencing data of 891 DNase-seq experiments from the ENCODE project [23] and identify the experiments corresponding to the collected cell lines. Given the raw sequencing data of a DNase-seq experiment, we define the chromatin accessibility score (*S*) of a DNA position as the number of reads (*N*) starting at this position divided by the average number of reads ($\tilde{N}$) starting at a position in a background region of size *W* centered at the given position [24], [25]. Formally, $S=N/\tilde{N}$ and $\tilde{N}=M/W$, where *M* is the number of reads starting within the background region. A summary of the data is shown in Table 1. The integration of DNA sequences and DNase-seq data not only enables the cell line-specific prediction but also effectively improves the performance of prediction (Figure S1).

**Table 1:** Summary of data.

**Cell line**	**No. of enhancers**	Sample*	**ID of DNase-seq experiment**
Epithelial cell of esophagus	148	15,188	ENCSR000ENN
Melanocyte	424	45,244	ENCSR518JGY
Cardiac fibroblast	446	49,656	ENCSR000ENH
Keratinocyte	497	54,343	ENCSR000EPQ
Myoblast	499	55,238	ENCSR000EOO
Stromal cell	710	81,295	ENCSR000EMH
Mesenchymal cell	1857	215,096	ENCSR405TXU
Natural killer cell	2677	281,512	ENCSR723JLG
Monocyte	7347	718,064	ENCSR000EPK

*Note*: *, number of positive samples after fixed-stride data augmentation (stride 1).

**Supplementary Figure S2:**
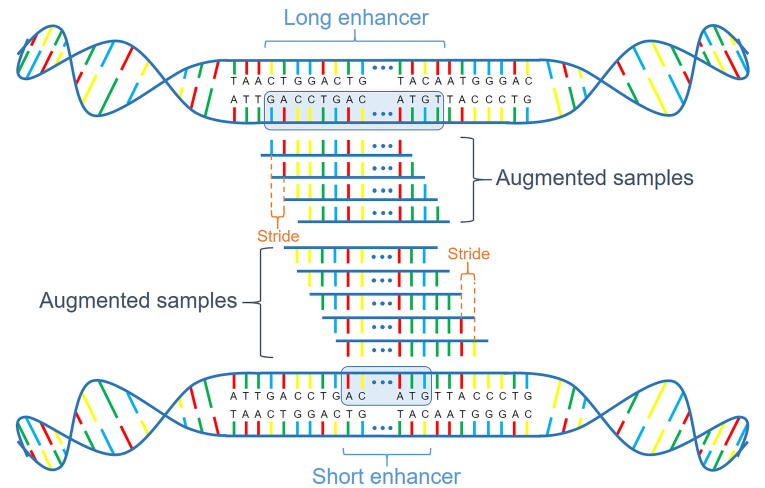
**Graphical illustration of the fixed-stride data augmentation strategy** Suppose that input fragments of fixed length *L* is required. When a fragment is longer than *L*, we slide a window of size *L* along the original sequence with stride s to obtain a number of sequences with length *L*. When a fragment is shorter than *L*, we slide a window of size *L* along the genome and take sequences overlapping with the original one.

We consider two issues that are crucial to our method. First, enhancers are of variable length, while a CNN requires inputs of fixed length. Second, a deep neural network has an appetite for a vast amount of training samples. We therefore propose a data augmentation strategy to address both issues (File S1, text A; Figure S2).

### Design of DeepCAPE

**Fig. 1:**
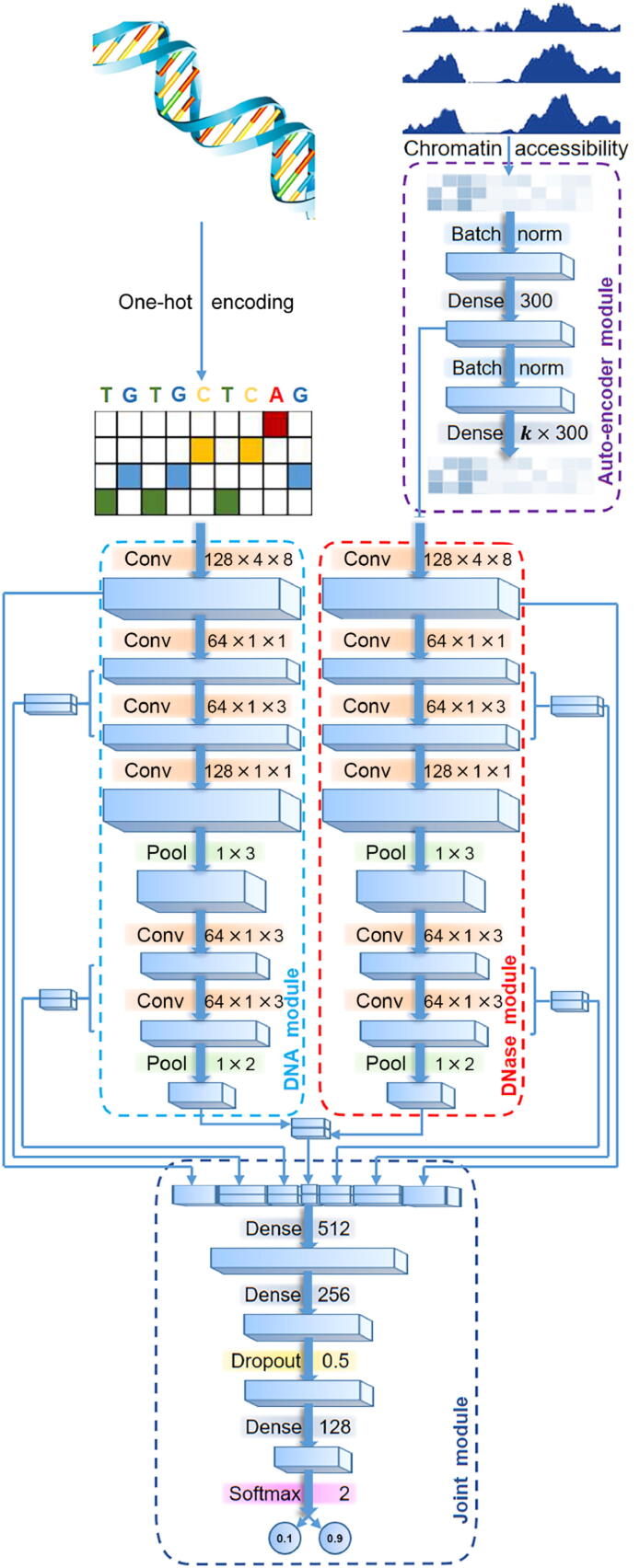
**Graphical illustration of DeepCAPE.** First, a DNA module is used to extract features of the input DNA fragment. Second, an auto-encoder module is adopted to embed DNase-seq data into a low-dimensional space. Third, a DNase module is used to extract features of chromatin accessibility after dimensionality reduction. Finally, a joint module integrates outputs of the DNA and DNase modules to predict the probability that an input sequence is an enhancer. Conv, convolutional layer; Pool, max-pooling layer; Batch norm, batch-normalization layer.

As illustrated in Fig. 1, DeepCAPE consists of four modules. First, a DNA module is used to extract features of DNA sequences. Second, an auto-encoder module is adopted to embed DNase-seq data into a low-dimensional space. Third, a DNase module is used to extract features of chromatin accessibility after dimensionality reduction. Finally, a joint module integrates outputs of the DNA and DNase modules to predict the probability that an input sequence is an enhancer.

#### DNA module

The DNA module is a CNN with multiple convolutional and pooling layers. The first layer uses 128 kernels to scan for sequence motifs of length 8 along the input DNA fragment, which is represented using the one-hot encoding. The second layer uses 64 kernels, each of length 1, to reduce the dimension of features extracted from the first layer by adopting the Network In Network (NIN) model [26], which aims to enhance the discrimination power of the model. The third layer uses 64 kernels, each of length 3, to reduce the number of parameters by drawing on experiences of VGGNet [27]. The fourth layer again adopts the NIN technique and uses 128 kernels, each of length 1, to extract high-level features. The fifth layer adopts the max-pooling strategy to reduce the number of parameters and abstract features learned in the previous layer. The sixth and seventh layers again adopt the VGGNet technique to further reduce the number of parameters by using 64 kernels, each of length 3. Finally, the eighth layer adopts the max-pooling strategy to abstract final high-level features. In the convolutional layers, the activation of the *k*-th convolutional kernel at the *i*-th position is written as.

$$a_{\mathrm{ik}}=ReLU\left(\sum_{m=0}^{M-1}\sum_{n=0}^{N-1}w_{\mathrm{mn}}^{k}x_{i+m,n}\right)\left(1\right)$$

where x is the input matrix, M is the size of the kernel, N is the number of input channels, and $w_{\mathrm{mn}}^{k}$ is the weight matrix of the kernel. For the first convolutional layer, N is equal to 4. For other layers, N is equal to the number of kernels in the previous layer. The rectified linear unit ReLU(x)=max(0,x) activation function sets negative values to zero. The aforementioned well-designed structure can effectively extract features of DNA sequences.

#### Auto-encoder and DNase modules

A DNase-seq experiment usually has a small number of replicates, and this number varies between cell lines, making the dimensionalities of input data variable between cell lines and preventing the use of a CNN in cross-cell line prediction. To solve this problem, we adopt auto-encoder, a neural network designed for unsupervised learning of efficient encodings [28], to embed the chromatin accessibility score of a DNA fragment into a vector of fixed length in a low-dimensional latent space. Briefly, the auto-encoder module first uses a batch-normalization layer to reduce the internal covariate shift and accelerate the training procedure. The output then goes to an encoder component, which is essentially a feedforward neural network that transfers the input data of k channels (corresponding to *k* replicates) into a single channel. After another batch-normalization layer, a decoder component, which is also a feedforward neural network, transfers the data back to *k* channels. With the module well trained, the decoder is able to produce output similar to the original input, and results of the encoder component can then be used as features extracted from the original data and fed to the successive DNase module. Such an auto-encoder module benefits our model in two aspects. First, regardless of the number of replicates for different cell lines, output of the module is of the same dimension, and thus makes cross-cell line prediction possible. Second, effective dimensionality reduction significantly alleviates the computational burden of the successive prediction model.

The DNase module extracts multi-level features from chromatin accessibility scores and is essentially identical to the DNA module in structure, except for the number of input channels. The DNA module is fed with one-hot encoded DNA sequence and has 4 channels, while the DNase module is fed with chromatin accessibility data produced by the encoder component of the auto-encoder module and has a single channel. A statistical analysis on a total of 43,011 experimentally verified enhancers in FANTOM shows that the median and mean lengths of these enhancers are 275 and 288 bp, respectively. We therefore select 300 as the dimensionality of the auto-encoder latent space, and thus the shapes of outputs of subsequent layers in DNA and DNase modules are symmetrical.

#### Joint module

The joint module integrates multi-level features from both the DNA and DNase modules to predict the probability that the input DNA fragment is an enhancer. Drawing on the idea of skip connection in ResNet [29], we merge outputs of the convolutional and max-pooling layers in DNA and DNase modules to form a multi-channel feedforward network. The merged outputs of different layers contain features of different levels, which are integrated via three fully connected hidden dense layers. Such a skip connection strategy endows the model the ability to self-adapt to different sizes of training sets. When there are sufficient training samples, the model may use low-level features. When there are inadequate training samples, the model inclines to explore high-level features automatically.

On the top of the architecture, a softmax layer predicts the probability that an input DNA fragment is an enhancer based on the integrated features, as.

$$f_{i}\left(z\right)=\frac{e^{z_{i}}}{\sum_{j}e^{z_{j}}}\left(2\right)$$

where $f_{i}\left(z\right)$ is the predicted probability that the input DNA fragment belongs to class i (*i.e.*, 1 for enhancer and 0 for non-enhancer).

### Model training

We carry out 5-fold cross-validation experiments to validate the performance of our method for each cell line. Particularly, in order to avoid information leakage, we partition both positive (known enhancers) and negative (non-enhancers) samples into 5 subsets of nearly equal size before converting sequences of variable length to sequences of fixed length by the data augmentation strategy. In each fold of the experiment, we take 4 subsets to train the model and test its performance using the remaining subset.

Considering that the positive and negative samples are highly imbalanced, we adopt a two-stage training strategy. First, we train an initial model using all positive samples and an equal number of negative samples sampled from the training set. After this stage, the DNA and DNase modules obtain the ability to extract features. Then, the joint module is further trained as usual using all the imbalanced samples on the training set, with learning rates of DNA and DNase modules setting to 0 [30]. This strategy also alleviates the computational burden. During training, the cross-entropy loss is adopted as the objective function to be optimized with Adam (File S1, text B).

With a well-trained model, we score all samples augmented from an original sequence on a test set, and then average over these scores to obtain the final probability that the sequence is an enhancer. We also used another strategy that takes the maximum of these scores as the final probability to study the effects of different statistics on the results.

We implement DeepCAPE in Python using Keras (https://keras.io) with Tensorflow as the backend, while the Theano backend also generated very close results according to our test. The NVIDIA GeForce GTX 1080Ti GPU is used to accelerate the computation. We have released our code in Github (https://github.com/ShengquanChen/DeepCAPE).

### Motif visualization

We propose a motif visualization strategy to interpret the features extracted by DeepCAPE. We convert kernels of the first convolutional layer to probabilistic position weight matrices (PWMs) by counting nucleotide occurrences in the set of sequences that activate the kernels. Briefly, each kernel of the first convolutional layer is converted into a PWM by scanning along input sequences for activated positions and then calculating the PWM by pooling corresponding regions [30], [31], [32]. A position i is regarded as being activated if.

$$\sum_{m=0}^{M-1}\sum_{n=0}^{N-1}w_{\mathrm{mn}}^{k}x_{i+m,n}>\alpha ∙EAV\left(3\right)$$

where α is the control coefficient (0<α<1) and EAV the extreme activation value defined as.

$$EAV=\sum_{m=0}^{M-1}{max(w}_{\mathrm{mn}}^{k}\left|0\leq n\leq N-1\right)\left(4\right)$$

We set length of kernels in the first convolutional layer to 8 and α to 0.9. We identify putative sequence motifs by using the tool TomTom 4.11.2 [33] with *q*-value threshold 0.1 to match PWMs identified by our method to the JASPAR database [34].

## Results

### DeepCAPE accurately predicts enhancers

To verify the performance of DeepCAPE, we conducted a series of 5-fold cross-validation experiments using enhancers collected from FANTOM and negative data generated by the background model with the consideration of GC content (see Method). We compared the performance of our method with several baseline methods, including gkmSVM [12], DeepSEA [17], and DeepEnhancer [18]. Using the same training and test sets with DeepCAPE, we retrained the three baseline methods with parameters or structures proposed by the respective authors and then evaluated their performance. We also proposed a variation of our model, named “DeepCAPE (seq only)”, which discarded the auto-encoder and DNase modules and predicted enhancers using only DNA sequence information. Considering our imbalanced classification task, we computed two widely used metrics, the area under the precision-recall curve (AUPRC) and the area under the receiver operating characteristic curve (AUROC).

**Fig. 2:**
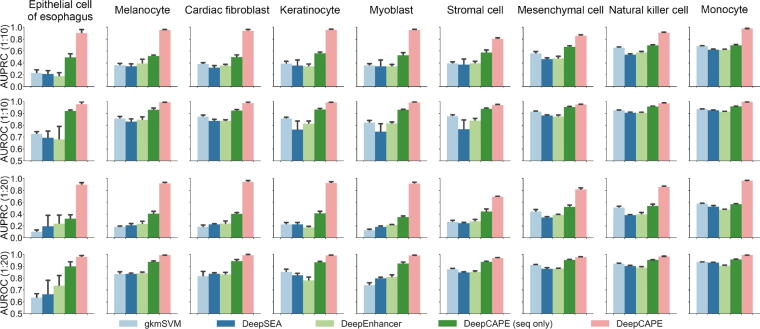
**Classification performance measured by AUPRC and AUROC at different ratios of positive to negative samples (1:10 and 1:20) with augmentation stride 1.** AUPRC, area under the precision-recall curve; AUROC, area under the receiver operating characteristic curve.

The performance at different ratios of positive to negative samples (1:10 and 1:20) with augmentation stride 1 is shown in Fig. 2. Our method consistently outperforms the three baseline methods. In more detail, when the ratios of positive to negative samples are 1:10 and 1:20, respectively, the AUPRC scores of our method are on average 0.474 and 0.590 higher than gkmSVM, 0.522 and 0.598 higher than DeepSEA, and 0.511 and 0.588 higher than DeepEnhancer. One-sided paired-sample Wilcoxon signed rank tests consistently suggest that our method achieves higher AUPRC scores than a baseline method (*P* < 2.2E − 16 for all the three baseline methods). In terms of AUROC scores, our method is on average 0.121 and 0.151 higher than gkmSVM, 0.169 and 0.151 higher than DeepSEA, and 0.150 and 0.150 higher than DeepEnhancer, when the ratios are 1:10 and 1:20, respectively. One-sided paired-sample Wilcoxon signed rank tests similar also consistently report significant results (*P* < 2.2E − 16 for all the three baseline methods). All these results suggest the superior performance of our method over existing sequence-based approaches in predicting enhancers.

We also compared the performance of our method to that of CENTIPEDE (with default parameters), which integrates DNA sequences and chromatin accessibility information to predict TFBSs [35]. With the same test data, DeepCAPE achieves a mean AUPRC of 0.919 and a mean AUROC of 0.985 for the 9 cell lines when the ratio of positive to negative samples is 1:10, while CENTIPEDE only achieves 0.760 and 0.826, respectively. Obviously, our method significantly outperforms CENTIPEDE (*P* < 2.2E − 16 for AUPRCs, *P* < 2.2E − 16 for AUROCs; one-sided paired-sample Wilcoxon signed rank test). Similarly, our method significantly outperforms CENTIPEDE when the ratio of positive to negative samples is 1:20 (*P* = 5.24E − 16 for AUPRCs and *P* < 2.2E − 16 for AUROCs; one-sided paired-sample Wilcoxon signed rank test).

We further validated the performance of our method on an independent test set with the following experiment [36]. Briefly, we trained our model using GM12878 enhancers downloaded from FANTOM (where enhancers are defined by CAGE tags) and corresponding genome background in chromosomes 1–15, and we tried to distinguish GM12878 enhancers downloaded from ENCODE (where enhancers are defined by computationally integrating ChIP-seq data) from corresponding background genome regions in the rest chromosomes. It is notable that, in this case, the source of enhancers is independent (CAGE or ChIP-seq), and there are not any overlaps between the training and test sets. Because of the massive number of enhancers in the dataset of GM12878 cell line, the most unbalanced dataset we can generate has the ratio 1:4 of positive to negative samples. In this case, our method achieves an AUPRC of 0.841 and an AUROC of 0.924, while CENTIPEDE only achieves 0.693 and 0.705, respectively. These results not only suggest that our method is capable of predicting enhancers in a context independent of the training data, but also demonstrate the superior performance of DeepCAPE over existing methods that integrate DNA sequences and chromatin accessibility information.

Our method demonstrates much higher robustness than the baseline methods. With the variance of AUPRCs in the 5-fold experiments calculated for each cell line, one-sided Wilcoxon rank sum tests consistently show that our method achieves smaller variance than a baseline method (*P* = 4.019E − 4 against gkmSVM, *P* = 7.908E − 4 against DeepSEA, and *P* = 4.571E − 3 against DeepEnhancer), suggesting that our method is not sensitive to the partition of training and test samples. Besides, our method consistently performs well in all the cell lines, while the performance of the other three methods shows significant fluctuation across cell lines, suggesting that they are sensitive to the number of training samples.

We further conducted a series of experiments to demonstrate the performance of DeepCAPE. Firstly, it is worth noting that the performance of the “DeepCAPE (seq only)” model is also superior to the three baseline methods in most cases, suggesting that our model has the advantage in the case of predicting only with sequences. Secondly, taking the maximum of the scores of samples augmented from an original test sequence as the final probability generates slightly worse performance, and this may be due to the outliers with high scores in the augmented negative samples. Finally, the performance on datasets without considering GC content is slightly superior to that on datasets under the GC content constraint (File S1, text C).

In terms of model training, benefiting from the usage of dropout layers and the early stop strategy, the performance on the test set is fairly close to that on the training set, indicating that DeepCAPE is able to avoid overfitting. In addition, with regard to the efficiency of model training, DeepCAPE is superior to other deep learning models due to the zero-learning-rate strategy in the second training stage. Take the dataset with augmentation stride 1 of myoblast as an example, when the ratio of positive to negative samples is 1:20, the training time for an epoch is about 126 s for DeepCAPE, 301 s for DeepSEA, and 237 s for DeepEnhancer.

### Contribution of each module

**Fig. 3:**
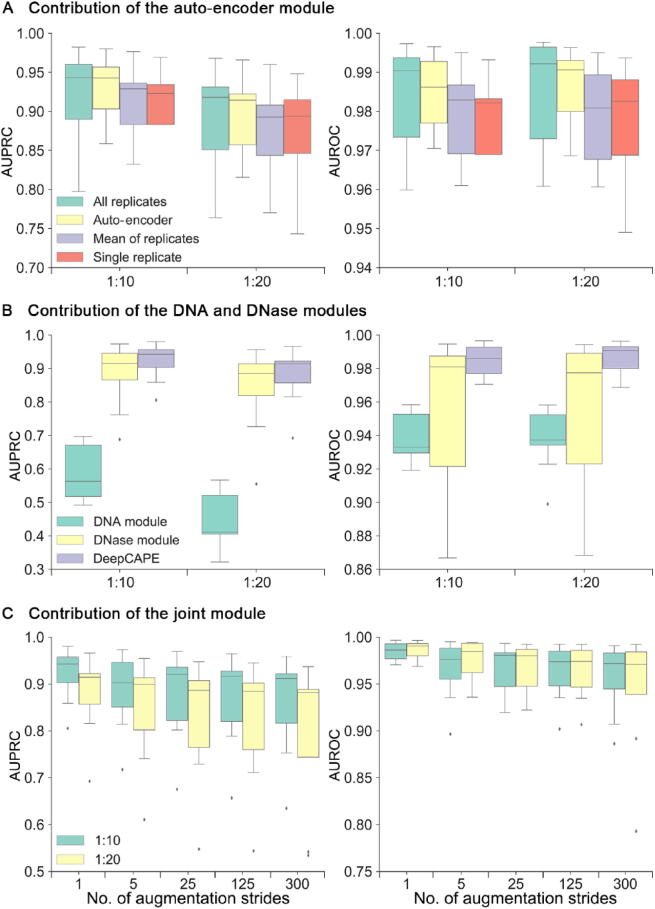
**Contribution of each module. A.** Performance of DeepCAPE with or without the auto-encoder module and other two strategies that average the replicates or randomly select a single replicate. **B.** Performance of DeepCAPE with either the DNA or DNase module. **C.** Performance of DeepCAPE on datasets of different numbers of augmentation strides.

To illustrate the contribution of auto-encoder module, we compared the performance of DeepCAPE with auto-encoder to that of DeepCAPE without auto-encoder as well as other two strategies that average the replicates or randomly select a single replicate. As shown in Fig. 3**A**, the auto-encoder module not only makes cross-cell line prediction possible, but also maintains the superior performance of our method even if the dimensionality of DNase-seq data is reduced (File S1, text D).

To evaluate contributions of the DNA and DNase modules, we performed a model ablation analysis. As shown in Fig. 3B, DNase-seq data provide more information to the prediction than DNA sequences and greatly improve the performance. In addition, jointly using DNA sequences and DNase-seq data effectively improves the performance and stability, indicating that DNA sequences also play an important role in promoting the performance of DeepCAPE and making the performance more stable (File S1, text E).

There are more than 100 million parameters in the whole neural network of DeepCAPE, and most of them are concentrated on the merge-layer of the joint module. As shown in Figure S1, we visualized activated features on the merge-layer when DeepCAPE was trained with datasets augmented by different strides. With abundant training samples, DeepCAPE is inclined to activate only low-level features, which are extracted by the first three layers. When the sample size is limited, however, DeepCAPE can also activate high-level features, which are extracted by the last three layers, indicating that DeepCAPE has the ability to self-adapt to different sizes of training sets (File S1, text F).

In order to explore the effect of the number of training samples to the final performance, we repeated the cross-validation experiments on datasets of different numbers of augmentation strides for each cell line. The results show that although the performance is decreasing overall with the increasing augmentation strides (Fig. 3C), the performance is still satisfactory when compared with the three baseline methods and the computational burden is significantly alleviated (File S1, text G; Table S3).

All the aforementioned observations suggest that DeepCAPE can not only achieve superior performance with limited known enhancers, but also achieve satisfactory performance with longer augmentation strides to effectively save computational time when there are massive enhancers.

### DeepCAPE enables cross-cell line prediction

Experimental approaches are expensive and time-consuming for large-scale identification of enhancers across a variety of human cell lines. For a cell line whose enhancers have not been identified yet, predicting potential enhancers has great significance in guiding biological experiments for novel enhancer identification.

To accurately predict enhancers across cell lines, we employed a collective scoring strategy. Given a cell line of interest and a DNA fragment, we used models trained on other cell lines to predict the probability that the fragment is an enhancer, and then averaged over these predictions to obtain a final prediction probability. The basic idea of this strategy is that a DNA sequence may be an enhancer in the new cell line if it plays a role of enhancer in some other cell lines. To better support the idea, we calculated the overlap rates between regions of enhancers of different cell lines. As shown in Table S1, the mean overlap rates of enhancers of most cell lines range from 15.4% to 21.8%, although those of natural killer cell and monocyte are 8.0% and 5.9%, respectively, due to the large number of enhancer samples in these two cell lines. In addition, we calculated the overlap rates between called DNase-seq peaks of different cell lines and found that the mean overlap rates range from 34.5% to 50.0% (Table S2). The results indicate that there are common enhancers and chromatin accessibility features in different cell lines, and thus we can utilize information from other cell lines to predict enhancers in a new cell line. To avoid introducing extra prior information, we directly averaged over the predictions from models of other cell lines to obtain a final prediction probability without other operation such as weighting different cell lines, thus making it easier to generalize the model.

We used the datasets of 9 cell lines from FANTOM to demonstrate the ability of DeepCAPE to predict enhancers in a cross-cell line manner. For each cell line, we first excluded the samples that overlap with samples in other cell lines to make sure that there are not common samples with other cell lines, thus making the task more challenging. On average, 35.2% and 37.6% of samples are left in the datasets of 9 cell lines when the ratios of positive to negative samples are 1:10 and 1:20, respectively, and the corresponding ratios become 1:8.3 and 1:18.1 averagely. We next used the models of other 8 cell lines to make predictions for the filtered samples of the cell line of interest, and then averaged over the resulting 8 probabilities to obtain the final prediction probability. We also used other three baseline models to predict enhancers in this cross-cell line manner.

**Fig. 4:**
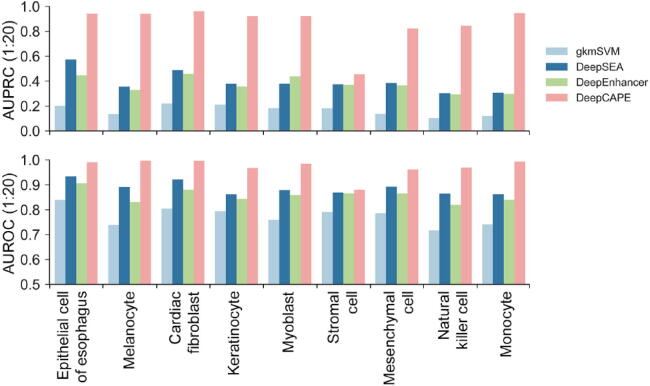
**Performance of the cross-cell line prediction strategy.** The cross-cell line prediction performance of DeepCAPE is consistently superior to other three baseline methods at the ratio of positive to negative samples (1:20).

**Supplementary Figure S3:**
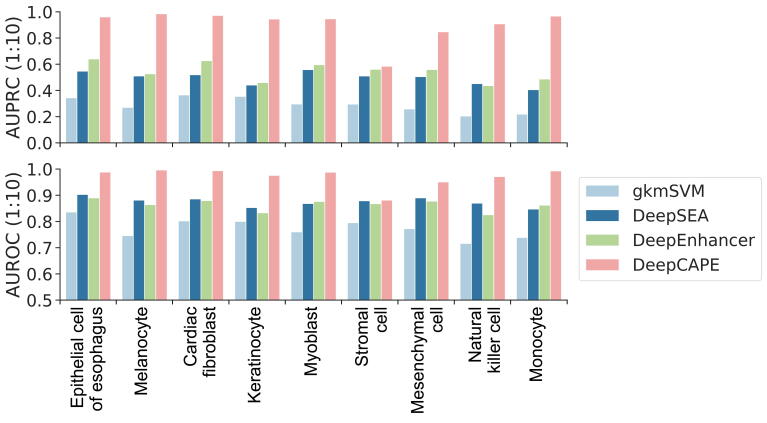
**Performance of the cross-cell line prediction strategy when the ratio of positive to negative samples is 1:10**

As shown in Fig. 4 and Figure S3, DeepCAPE with our cross-cell line prediction strategy is consistently superior to other three baseline methods. In more detail, when the ratio of positive to negative samples is 1:10, the average AUPRC and AUROC scores of DeepCAPE in 9 cell lines are 0.902 and 0.971, respectively; when the ratio is 1:20, the average AUPRC and AUROC scores are 0.862 and 0.971, respectively. These results suggest that DeepCAPE can accurately predict enhancers across cell lines and thus establish a landscape of potential enhancers specific to a cell line that still lacks systematic exploration of enhancers. The relatively low performance on the dataset of stromal cell may be caused by the fact that we can find only DNase-seq data of stromal cell of bone marrow in ENCODE, which may not match the cell line in FANTOM very well.

### DeepCAPE recovers known TF binding motifs

To interpret features extracted by DeepCAPE, we used a motif visualization strategy (see Method) to obtain sequence signatures (*i.e.*, PWMs) learned from the first convolutional layer of the DNA module. We further identified putative motifs by using the tool TomTom [33] to match these PWMs to the JASPAR database [34].

**Fig. 5:**
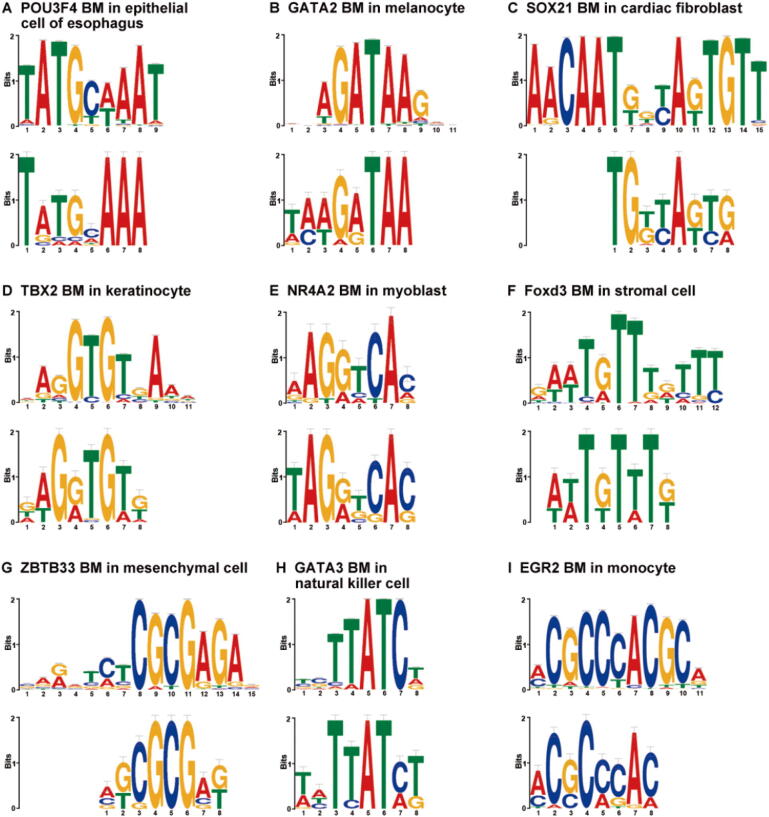
**Visualization of TF binding motifs learned by DeepCAPE from kernels of the first convolutional layer.** In each panel, a known TF BM from the JASPAR database is shown on the top, while the motif learned by DeepCAPE is shown at the bottom. TF, transcription factor; BM, binding motif.

For each cell line, we displayed the sequence logo of one of the matched motifs in Fig. 5. In the dataset of cardiac fibroblast, DeepCAPE recovers a binding motif (BM) of SOX21, whose ectopic expression in embryonic stem cells induces their differentiation into specific cell types, including those that express markers representative of heart development [37]. In the dataset of keratinocyte, DeepCAPE recovers a BM of TBX2, which represses the transcription from the long control region of human papillomaviruses [38]. In the dataset of myoblast, DeepCAPE recovers a BM of NR4A2, which has been previously shown to contain consensus cAMP response element binding protein (CREB) binding sites that are occupied by CREB and phospho-CREB in myoblasts [39]. In the dataset of natural killer cell, DeepCAPE recovers a BM of GATA3, which is a critical regulator for natural killer cell terminal maturation [40]. In the dataset of monocyte, DeepCAPE recovers a BM of EGR2, which shows prominent, transient induction in β-glucan-exposed monocytes [41]. It has been demonstrated that enhancers with EGR2 motifs are mainly associated with genes involved in lipid metabolism and biosynthesis and lysosome function [41]. To sum up, DeepCAPE can help us find potential TF binding in specific cell lines.

### Applications of DeepCAPE

**Fig. 6:**
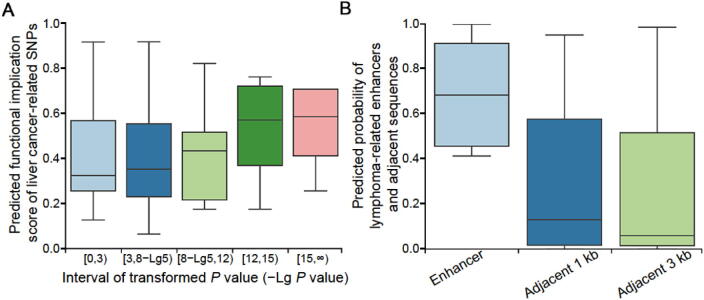
**Applications of DeepCAPE. A.** The distributions of predicted functional implication scores of the liver cancer-related SNPs according to different intervals of transformed *P* value (−Lg *P* value). **B.** The distributions of predicted probabilities of the lymphoma-related enhancers and their adjacent sequences sampled from either 1-kb or 3-kb upstream and downstream regions. SNP, single nucleotide polymorphism.

To demonstrate potential applications of DeepCAPE, we collected 334 single nucleotide polymorphisms (SNPs) that were possibly associated with liver cancer from GRASP [42]. Each SNP has an association *P* value obtained from a GWAS regarding liver cancer. We identified a liver cancer cell line (HepG2) in ENCODE and trained a DeepCAPE model using enhancers and DNase-seq data specific to this cell line. We then calculated a probability that indicates whether a DNA fragment of 300 bp surrounding a SNP is an enhancer for each of the 334 SNPs. We finally classified the SNPs into 5 groups according to log_10_-transformed *P* values of the SNPs and drew box plots of the predicted probabilities for each group. As shown in Fig. 6**A**, the predicted probabilities for SNPs with smaller *P* values are relatively higher than those with larger *P* values. This observation suggests that predictions given by our method using genomic and epigenomic data are potentially correlated with *P* values obtained from genetic studies.

We further collected 14 enhancers that are shown to be associated with lymphoma from the literature [43] and showed the ability of our method to discriminant these enhancers from their nearby DNA fragments. For this purpose, we first used enhancers and DNase-seq data specific to a lymphocyte cell line (GM12878) in ENCODE to train a DeepCAPE model. We then used this model to calculate prediction probabilities for the 14 lymphoma-related enhancers and the same number of their adjacent sequences sampled from either 1-kb or 3-kb upstream and downstream regions. We drew box plots of the predicted probabilities in Fig. 6B. It is obvious that prediction probabilities of the lymphoma-related enhancers are significantly higher than those of the adjacent sequences (*P* = 7.915E − 6 for adjacent 1 kb, *P* = 9.032E − 7 for adjacent 3 kb; one-sided paired-sample Wilcoxon signed rank test). These results suggest that our method has the potential ability to discriminant enhancers related to lymphoma from their nearby DNA fragments.

## Discussion

We have introduced a deep learning framework named DeepCAPE to integrate DNA sequence information and chromatin accessibility data for predicting enhancers. Benefitting from the integration of DNase-seq data, the well-designed feature extraction modules, the skip connection strategy, and the adoption of auto-encoder, DeepCAPE is superior to existing methods in the imbalanced classification of cell line-specific enhancers against background sequences, self-adaptable to different sizes of datasets, capable of making cross-cell line predictions, and interpretable in extracted features. We have further demonstrated the potential ability of DeepCAPE to explain functional implications of genetic variants and discriminate disease-related enhancers. Our method has two main application scenarios. Firstly, one can use our method to establish a landscape of potential enhancers specific to a cell line that still lacks systematic exploration of enhancers, thereby promoting the deciphering of regulatory mechanisms for the cell line. Secondly, one can use our method to explore functional implications of genetic variants or DNA fragments specific to a cell line, thereby bridging genomic and genetic studies toward the understanding of disease development.

Certainly, our work can further be improved in several aspects. Firstly, the incorporation of the long short-term memory (LSTM) network, a kind of recurrent neural network architectures, into our framework may further improve the performance, because LSTM may be able to capture very long-range interaction in the sequence. In addition, the adaptation of an embedding representation of DNA sequences instead of the use of the one-hot encoding may also benefit the prediction accuracy [44]. Secondly, since we have shown that the first convolutional layer is an effective motif discoverer, researchers may use our model to learn the complex grammar of TF binding in specific cell lines. In addition, one can also explore interactions of motifs in higher convolutional layers. Thirdly, the inclusion of other epigenetic features like methylation and histone modifications may further improve the performance. Considering it is costly to obtain such experimental data, we can further include other epigenetic features in the future. Fourthly, the definition of negative samples can be further improved in future work. For example, a technique, Annotating Genes with Positive Samples (AGPS), refines the negative set in an iterative manner [45]; a hybrid sampling algorithm, which integrates both ensemble classifier and over-sampling techniques, is proposed to deal with imbalanced data [46]. Fifthly, we can further study the pathways possibly affected by the predicted enhancers [47]. Sixthly, our deep learning framework can possibly be used to identify other functional elements and model gene regulation [48], [49], [50]. Seventhly, our data integration framework can possibly be adapted for the characterization of single-cell chromatin accessibility sequencing data [51], [52], [53]. Finally, our framework can also be generalized for the prediction of functional impacts of genomic mutations and the prioritization of candidate variants in whole-genome sequencing studies, thereby facilitating both research and practice of precision medicine [54].

## Code availability

The source code and detailed tutorial of DeepCAPE are freely available at https://github.com/ShengquanChen/DeepCAPE.

## Competing interests

The authors have declared no competing interests.

### CRediT authorship contribution statement

**Shengquan Chen:** Data curation, Formal analysis, Investigation, Methodology, Writing – original draft. **Mingxin Gan:** Conceptualization, Funding acquisition, Project administration. **Hairong Lv:** Data curation, Validation, Methodology. **Rui Jiang:** Conceptualization, Funding acquisition, Project administration, Writing – review & editing.

## Supplementary material

Supplementary File S1 **Supplementary texts for Method and Results**
