# Supplementary material for: DeepCAPE: A Deep Convolutional Neural Network for the Accurate Prediction of Enhancers
Source: Genomics Proteomics Bioinformatics. 2021 Feb 11;19(4):565–77. doi: 10.1016/j.gpb.2019.04.006 (PMC9040020; doi:10.1016/j.gpb.2019.04.006)
Supplement: Supplementary File S1 — Supplementary texts for Method and Results [file mmc1.docx]

**File S1 Supplementary texts for Method and Results**

**Supplementary text A: data augmentation strategies**

We proposed a “fixed-stride” data augmentation strategy as illustrated in Figure S2. Suppose that the CNN model requires input fragments of length *L*. In the case that a fragment is longer than *L*, we slide a window of size *L* along the original sequence with stride *s* to obtain a number of sequences of length *L*. In the case that an fragment is shorter than *L*, we slide a window of size *L* along the genome and take sequences overlapping with the original one to obtain augmented sequences. A statistical analysis on a total of 43,011 experimentally veriﬁed enhancers in FANTOM shows that the median and mean length of these enhancers are 275 and 288 bps, respectively, and hence we set *L* to 300. We can further control the number of augmented sequences by changing the value of stride *s*. With this strategy, input sequences of variable lengths are converted into ﬁxed-length samples, and the number of available training samples increases greatly. We also propose an alternative “fixed-ratio” augmentation strategy as follows. Given an original sequence of length *X* and a pre-defined augmentation ratio *r*, we slide a window of size *L* with a derived stride (*L* + *X*)/*r* along the genome to obtain *r* sequences overlapping with the original one. The fixed-stride strategy is used throughout this paper, and the fixed-ratio strategy is used to show the robustness of our model to different data augmentation strategies.

**Supplementary text B: model training**

During training, the cross-entropy loss, defined as the entropy between a true distribution $p$ and the estimated class probabilities $q$, is adopted as the objective function to be optimized, as

$$H\left( p,q \right)=-\sum_{x} p\left( x \right)\log q\left( x \right).$$

We adopt Adam, a widely used algorithm for first-order gradient-based optimization of stochastic objective functions [1], to optimize the objective function, with the initial learning rate set to 10^-4^. The learning rate decay schedule and the early stopping strategy are adopted to accelerate the convergence of training and avoid overfitting. Batch-normalization layers are used to accelerate training by reducing the internal covariate shift [2]. A dropout layer is used between the last two fully connected layers, and it randomly drops half of the units to avoid overfitting [3].

**Supplementary text C: experiments to demonstrate the performance of DeepCAPE**

We conducted a series of experiments to further illustrate the performance of DeepCAPE. Firstly, it is worth noting that the performance of the “seq only” model, which only uses DNA sequence information, is also superior to the three baseline methods and performs more steadily, suggesting that our model has the advantage in the case of predicting only with sequences. Secondly, the “fixed-ratio” data augmentation strategy does not significantly influence the performance of our method. For example, the fixed-stride strategy with stride 1 and the fixed-ratio strategy with ratio 100 produced similar number of augmented sequences, and two-sided paired-sample Wilcoxon tests suggestted that their performance were not significantly different (*P* value = 0.7972, 0.2304, 0.6999, and 0.161 for area under the precision-recall curve (auPRC, 1:10), area under the receiver operating characteristic curve (auROC, 1:10), auPRC (1:20) and auROC (1:20), respectively). Thirdly, taking the maximum of the scores of sequences augmented from an original test sample as the final probability generated slightly worse performance with the mean auPRC and auROC decreased by 0.051 and 0.011 respectively when the ratio of positive and negative samples was 1:20. The decline in performance might be due to the outliers with high scores in the augmented negative samples. Finally, we also repeated the prediction experiments with datasets generated by a less rigorous background model without considering GC content to demonstrate the adaptability of our method to different genome contexts. The performance on datasets without considering GC content was slightly superior to that on datasets under the GC content constraint with averagely improved auPRC of 0.041 and auROC of 0.003 when the ratio of positive and negative samples was 1:20.

**Supplementary text D: contribution of auto-encoder module**

Dimensionality reduction of chromatin accessibility scores enabled by the auto-encoder module significantly reduces the amount of data and alleviates the computational burden. To prove that the data after dimensionality reduction is still informative, we repeated for each cell line the 5-fold cross-validation experiments with the auto-encoder module excluded (and thus the dimensions of input data for the DNase-module were different and cross cell-line prediction did not work). As shown in **Figure 3**A, the auto-encoder module did not significantly influence the performance (two-sided paired-sample Wilcoxon test *P* value = 0.5813) but slightly improved the stability of the results. These observations suggest that the auto-encoder module not only makes cross cell-line prediction possible, but also maintains the superior performance of our method even if the dimensionality of the DNase-seq data is reduced. We also compared the performance of models with auto-encoder to other two strategies that average the replicates or randomly select a single replicate. As shown in Figure 3A, the performance of models using auto-encoder was superior to that of averaging the replicates with 2.63% and 3.06% improvement of auPRCs when the ratios of positive and negative samples were 1:10 and 1:20, respectively, and that are 3.59% and 3.56% to the performance of randomly selecting a single replicate.

**Supplementary text E: contributions of DNA and DNase modules**

We performed a model ablation analysis by repeating the cross-validation experiments with either the DNA or DNase module excluded to evaluate contributions of these modules. As shown in Figure 3B, there were evident differences in the contributions of the DNA and DNase modules, especially in terms of the auPRC. After removing the DNA module, the mean auPRCs decreased by 4.64% and 5.84% when the ratios of positive and negative samples were 1:10 and 1:20, respectively. When removing the DNase module, however, the mean auPRCs dropped by 36.39% and 49.38%, respectively. Obviously, DNase-seq data provide more information than DNA sequences to accurately predict enhancers. In addition, using DNA sequences and DNase-seq data jointly effectively improves performance and stability, indicating that DNA sequences also play an important role in promoting the performance of DeepCAPE and making the performance more stable.

**Supplementary text F: DeepCAPE can self-adapt to different sizes of datasets**

As shown in **Table 1** and **Figure 2**, with even only a few thousand training samples, DeepCAPE still performed very well in all the 9 cell lines, while the performance of the three baseline methods was greatly affected by the number of training samples. This means that our method can automatically adapt to different sizes of training sets for better performance and thus achieve superior performance on a dataset with limited number of known enhancers.

We further visualized activated features on the merge-layer of the joint module when DeepCAPE was trained with datasets augmented by different strides. With a model trained, we fed positive and negative samples to the network, calculated values of features for each sample, and defined the activation degree of a feature as the absolute difference of its values between positive and negative samples. Taking the cell line of myoblast as an example, we plotted heat-maps of activation degrees for features coming from different convolutional and pooling layers in both the DNA and DNase modules in Figure S1. Briefly, with abundant training samples (*e.g.*, stride 1), DeepCAPE was inclined to activate only low-level features, which were extracted by the first three layers. When the sample size was limited (*e.g.*, stride 300), however, DeepCAPE can also activated high-level features, which were extracted by the last three layers.

**Supplementary text G: DeepCAPE achieves satisfactory performance with limit datasets to effectively save computational time**

The massive training samples are helpful to improve the prediction performance of our method. However, it may not be necessary to use all the samples for training with the consideration of the computational burden. In order to explore the effect of the number of training samples to the final performance, we repeated the cross-validation experiments on datasets of different augmentation strides for each cell line. As shown in Figure 3 (C), although the performance was gradually decreasing with the augmentation stride becomes longer, the performance was still satisfactory when compared with the three baseline methods. In more detail, we reported in Table S3 the mean auPRC of DeepCAPE in each cell line with different augmentation strides and corresponding time consumed in each training epoch when the ratio of positive and negative samples was 1:20. When the stride was 5, the mean auPRC decreased only 4.026%, while 79.753% of the computational time was saved. More extremely, when the stride was 25, the mean auPRC decreased only 6.542% but 96.620% of the computational time was saved, indicating that DeepCAPE can achieve satisfactory performance with longer augmentation strides to effectively save computational time when there are massive enhancer samples.

**References**

[1] Kingma DP, Ba J. Adam: a method for stochastic optimization. arXiv preprint arXiv 2014; 1412.6980.

[2] Ioffe S, Szegedy C. Batch normalization: accelerating deep network training by reducing internal covariate shift. arXiv preprint arXiv 2015;1502.03167.

[3] Srivastava N, Hinton G, Krizhevsky A, Sutskever I, Salakhutdinov R. Dropout: a simple way to prevent neural networks from overfitting. J Mach Learn Res 2014;15:1929−58.
