# Supplementary material for: DeepCAPE: A Deep Convolutional Neural Network for the Accurate Prediction of Enhancers
Source: Genomics Proteomics Bioinformatics. 2021 Feb 11;19(4):565–77. doi: 10.1016/j.gpb.2019.04.006 (PMC9040020; doi:10.1016/j.gpb.2019.04.006)
Supplement: Supplementary Table S1 [file mmc2.docx]

**Table S1 The overlap rates between regions of enhancers of different cell lines**

|  | **Epithelial cell** | **Melanocyte** | **Cardiac fibroblast** | **Keratinocyte** | **Myoblast** | **Stromal cell** | **Mesenchymal cell** | **Natural killer cell** | **Monocyte** |
| --- | --- | --- | --- | --- | --- | --- | --- | --- | --- |
| Epithelial cell | - | 3.0% | 8.0% | 11.0% | 3.0% | 6.0% | 2.0% | 0.0% | 1.0% |
| Melanocyte | 9.0% | - | 11.0% | 10.0% | 10.0% | 9.0% | 6.0% | 1.0% | 3.0% |
| Cardiac fibroblast | 25.0% | 11.0% | - | 19.0% | 15.0% | 20.0% | 9.0% | 1.0% | 2.0% |
| Keratinocyte | 39.0% | 12.0% | 21.0% | - | 13.0% | 16.0% | 7.0% | 1.0% | 2.0% |
| Myoblast | 11.0% | 11.0% | 17.0% | 13.0% | - | 17.0% | 9.0% | 1.0% | 2.0% |
| Stromal cell | 30.0% | 16.0% | 32.0% | 22.0% | 24.0% | - | 14.0% | 2.0% | 5.0% |
| Mesenchymal cell | 30.0% | 26.0% | 36.0% | 26.0% | 35.0% | 37.0% | - | 12.0% | 15.0% |
| Natural killer cell | 4.0% | 7.0% | 4.0% | 8.0% | 6.0% | 9.0% | 18.0% | - | 17.0% |
| Monocyte | 26.0% | 49.0% | 32.0% | 34.0% | 30.0% | 47.0% | 58.0% | 46.0% | - |
| Mean | 21.8% | 16.9% | 20.1% | 17.9% | 17.0% | 20.1% | 15.4% | 8.0% | 5.9% |

*Note*: Each column means the overlap rates between regions of enhancers of the specific cell line and other cell lines in the rows.
