# Supplementary material for: DeepCAPE: A Deep Convolutional Neural Network for the Accurate Prediction of Enhancers
Source: Genomics Proteomics Bioinformatics. 2021 Feb 11;19(4):565–77. doi: 10.1016/j.gpb.2019.04.006 (PMC9040020; doi:10.1016/j.gpb.2019.04.006)
Supplement: Supplementary Table S2 [file mmc3.docx]

**Table S2 The overlap rates between called DNase-seq peaks of different cell lines**

|  | **Epithelial cell** | **Melanocyte** | **Cardiac fibroblast** | **Keratinocyte** | **Myoblast** | **Stromal cell** | **Mesenchymal cell** | **Natural killer cell** | **Monocyte** |
| --- | --- | --- | --- | --- | --- | --- | --- | --- | --- |
| Epithelial cell | - | 40.0% | 51.0% | 75.0% | 41.0% | 46.0% | 48.0% | 53.0% | 56.0% |
| Melanocyte | 30.0% | - | 40.0% | 35.0% | 29.0% | 34.0% | 37.0% | 51.0% | 45.0% |
| Cardiac fibroblast | 42.0% | 44.0% | - | 46.0% | 47.0% | 56.0% | 57.0% | 53.0% | 54.0% |
| Keratinocyte | 52.0% | 32.0% | 38.0% | - | 30.0% | 33.0% | 35.0% | 47.0% | 45.0% |
| Myoblast | 45.0% | 42.0% | 61.0% | 48.0% | - | 59.0% | 56.0% | 46.0% | 51.0% |
| Stromal cell | 49.0% | 48.0% | 72.0% | 51.0% | 57.0% | - | 65.0% | 54.0% | 58.0% |
| Mesenchymal cell | 42.0% | 43.0% | 60.0% | 44.0% | 45.0% | 53.0% | - | 55.0% | 50.0% |
| Natural killer cell | 15.0% | 18.0% | 17.0% | 18.0% | 11.0% | 14.0% | 17.0% | - | 33.0% |
| Monocyte | 19.0% | 20.0% | 22.0% | 22.0% | 16.0% | 18.0% | 20.0% | 41.0% | - |
| Mean | 36.8% | 35.9% | 45.1% | 42.4% | 34.5% | 39.1% | 41.9% | 50.0% | 49.0% |

*Note*: Each column means the overlap rates between called DNase-seq peaks of the specific cell line and other cell lines in the rows.
