# Supplementary material for: DeepCAPE: A Deep Convolutional Neural Network for the Accurate Prediction of Enhancers
Source: Genomics Proteomics Bioinformatics. 2021 Feb 11;19(4):565–77. doi: 10.1016/j.gpb.2019.04.006 (PMC9040020; doi:10.1016/j.gpb.2019.04.006)
Supplement: Supplementary Table S3 [file mmc4.docx]

**Table S3 The mean AUPRC of DeepCAPE in each cell line with different augmentation strides and corresponding time consumed in each training epoch when the ratio of positive to negative samples is 1:20**

| **Cell line** | **Stride** | | | | | | | |
| --- | --- | --- | --- | --- | --- | --- | --- | --- |
|  | **1** | |  | **5** | |  | **25** | |
|  | **AUPRC** | **Time (s)** |  | **AUPRC** | **Time (s)** |  | **AUPRC** | **Time (s)** |
| Epithelial cell of esophagus | 0.896 | 37 |  | 0.874 | 7 |  | 0.857 | 1 |
| Melanocyte | 0.919 | 95 |  | 0.913 | 20 |  | 0.911 | 3 |
| Cardiac fibroblast | 0.943 | 105 |  | 0.931 | 23 |  | 0.907 | 4 |
| Keratinocyte | 0.922 | 121 |  | 0.899 | 25 |  | 0.897 | 4 |
| Myoblast | 0.914 | 126 |  | 0.908 | 25 |  | 0.887 | 4 |
| Stromal cell | 0.692 | 175 |  | 0.610 | 36 |  | 0.548 | 7 |
| Mesenchymal cell | 0.816 | 475 |  | 0.741 | 89 |  | 0.729 | 17 |
| Natural killer cell | 0.857 | 617 |  | 0.802 | 126 |  | 0.765 | 22 |
| Monocyte | 0.966 | 1631 |  | 0.954 | 328 |  | 0.947 | 51 |
